# Primary Mammary Angiosarcoma: Literature Review

**DOI:** 10.7759/cureus.8589

**Published:** 2020-06-12

**Authors:** Dhirendra Nath Soren, Gopalakrishnan Gunasekaran, Debasis Naik, Gouranga Charan Prusty, Sakthivel Chinnakkulam Kandhasamy

**Affiliations:** 1 General Surgery, Veer Surendra Sai Institute of Medical Sciences and Research, Burla, IND; 2 Surgical Gastroenterology, Jawaharlal Institute of Postgraduate Medical Education and Research, Puducherry, IND; 3 General Surgery, Jawaharlal Institute of Postgraduate Medical Education and Research, Puducherry, IND; 4 Pathology, Pandit Raghunath Murmu Medical College and Hospital, Baripada, IND; 5 Surgery, Jawaharlal Institute of Postgraduate Medical Education and Research, Puducherry, IND

**Keywords:** primary mammary angiosarcoma, cutaneous angiosarcoma, blood lakes, cd31, cd34, wide local excision, mastectomy, factor viii

## Abstract

Angiosarcomas of the breast are extremely rare, highly aggressive tumors of vascular origin comprising 0.04% of all malignant neoplasms of the breast. They can be classified into primary mammary angiosarcomas and cutaneous (secondary) angiosarcomas. Primary angiosarcomas, owing to their unusual clinical presentation, are diagnosed late. In addition, the available literature to date lacks sufficient evidence to establish standard treatment guidelines for this group of tumors, thereby resulting in poor prognosis. In medical database, most available papers concern secondary angiosarcomas, with only a few case reports of primary angiosarcomas. The aim of this paper is to review what is known hitherto about the presentation, diagnostic tools, and therapeutic modalities for primary mammary angiosarcomas.

## Introduction and background

Primary angiosarcomas of the breast are extremely rare, highly aggressive tumors of vascular origin accounting for 0.04% of all malignant neoplasms of the breast [1]. Angiosarcomas of the breast can be classified into primary angiosarcomas, arising de novo, and cutaneous (secondary) angiosarcomas, developing as a consequence of previous breast cancer treatment (e.g., prior postoperative radiotherapy and/or long-standing lymphedema following treatment for breast cancer, known as Stewart-Treves syndrome). Primary mammary angiosarcomas arise within the breast parenchyma with infrequent invasion of the skin [2]. Owing to its unusual clinical presentation and rarity, the diagnosis is usually delayed, thereby resulting in a poorer prognosis. In addition, in medical databases, most available articles report secondary angiosarcomas, with only a few case reports of primary mammary angiosarcomas. We therefore attempt to review the literature, which will help in clarifying what is known about primary breast angiosarcomas hitherto.

## Review

Epidemiology

Mammary angiosarcomas are extremely rare, with malignant fibrous histiocytoma, fibrosarcoma, and liposarcoma being the more common histological subtypes of breast sarcomas. Only around 20% of the mammary angiosarcomas are primary angiosarcomas, with an incidence of around 17 new cases per million women [3].

Etiology

Etiology is unknown. This malignant tumor occurs primarily in young women, with 6-12% of the cases occurring while they are gestating, suggesting a hormonal effect [4,5]. Nonetheless, estrogen receptor positivity is so uncommon in the reported cases that the hormonal dependency of angiosarcoma is still undetermined [6].

Clinical presentation

**Figure 1:**
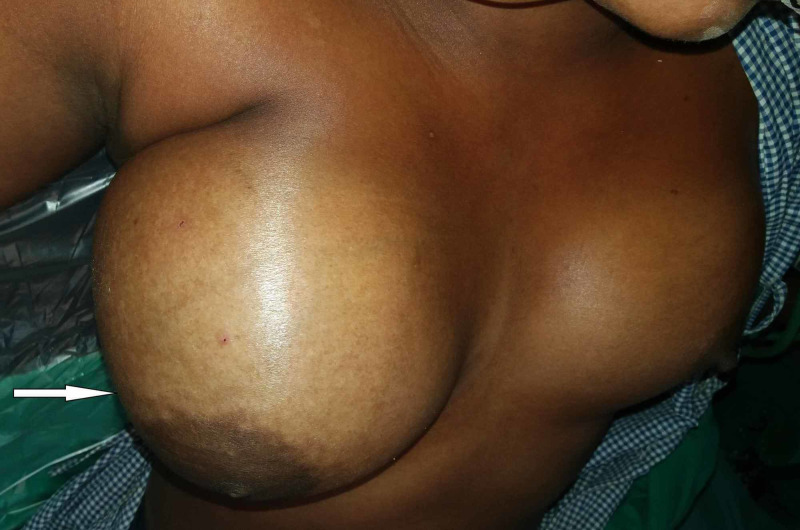
Clinical presentation of mammary angiosarcoma, presenting with a rapidly increasing painless breast mass.

Primary angiosarcomas are usually observed in women in the third to fifth decade of life, presenting either with a sense of fullness of breast or an insidious onset, rapidly growing painless palpable mass (Figure 1). Tumors that are large and superficial often present with purplish-blue discoloration of the overlying skin [7-9]. Large masses have also been reported, steering the consumption of platelets and clotting factors and the hemorrhagic manifestations of hemangioma thrombocytopenia syndrome [10,11].

Diagnosis

Though there is some evidence that mammography and ultrasonography may raise a suspicion, there are no specific pathognomonic features that can point toward this diagnosis. Mammographic findings include an ill-defined mass without calcification with associated overlying skin thickening. Ultrasound may reveal a heterogeneous mass, with hypervascularity demonstrable on color Doppler [9]. MRI is the radiological investigation of choice for characterizing and diagnosing breast angiosarcomas. This is due to its characteristic ability of proper evaluation of the regional extent of the tumor and envisioning the vascular nature of the lesion. The tumor appears as a heterogeneous mass with hypointensity on T1-weighted and hyperintensity on T2-weighted images, with high-grade tumors usually exhibiting swift enhancement with washout kinetics and lower grade tumors exhibiting persistent or plateau enhancement kinetics. High-grade tumors can also exhibit localized areas of hyperintensity on T1-weighted images, reflecting hemorrhage or vascular lakes [12]. In addition, CT of the thorax may be useful to look for lung metastasis.

**Figure 2:**
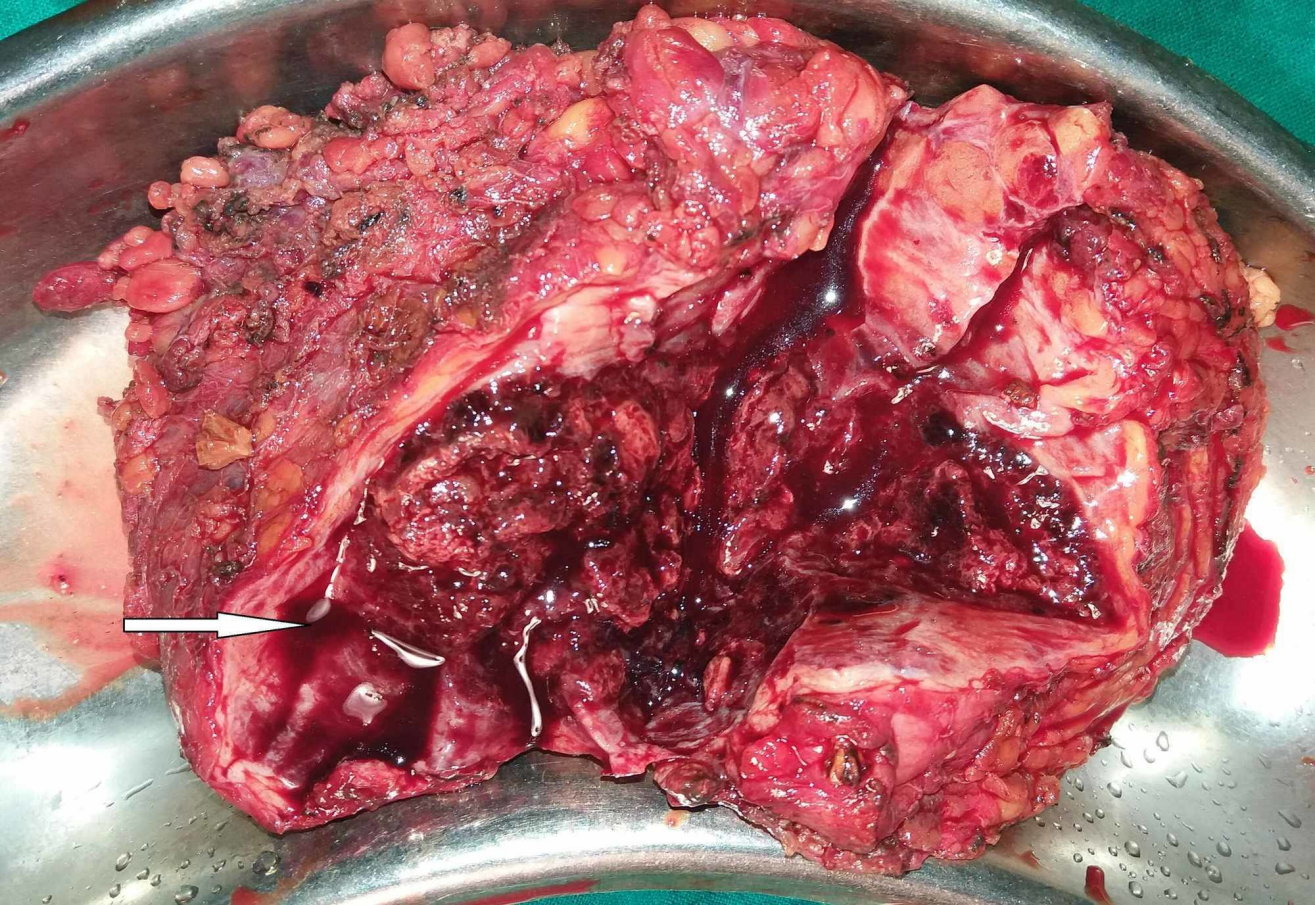
Gross specimen following wide local excision showing a lobulated mass, cut section of which shows areas of hemorrhage.

**Figure 3:**
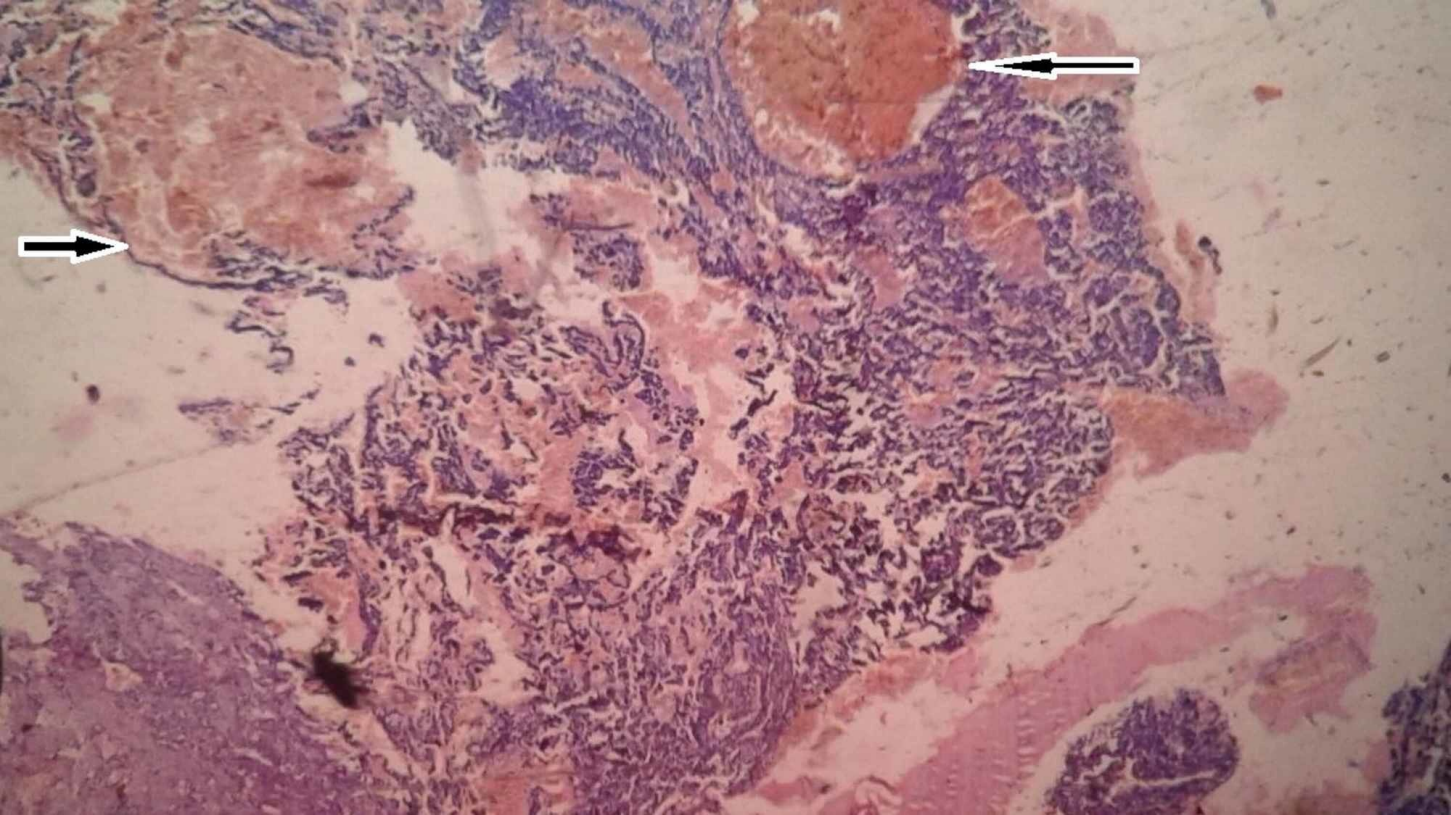
Histopathology showing the presence of dilated and thrombosed blood vessels surrounded by neoplastic cells with areas of hemorrhage (blood lakes) (100x, H&E). H&E, hematoxylin and eosin stain

**Figure 4:**
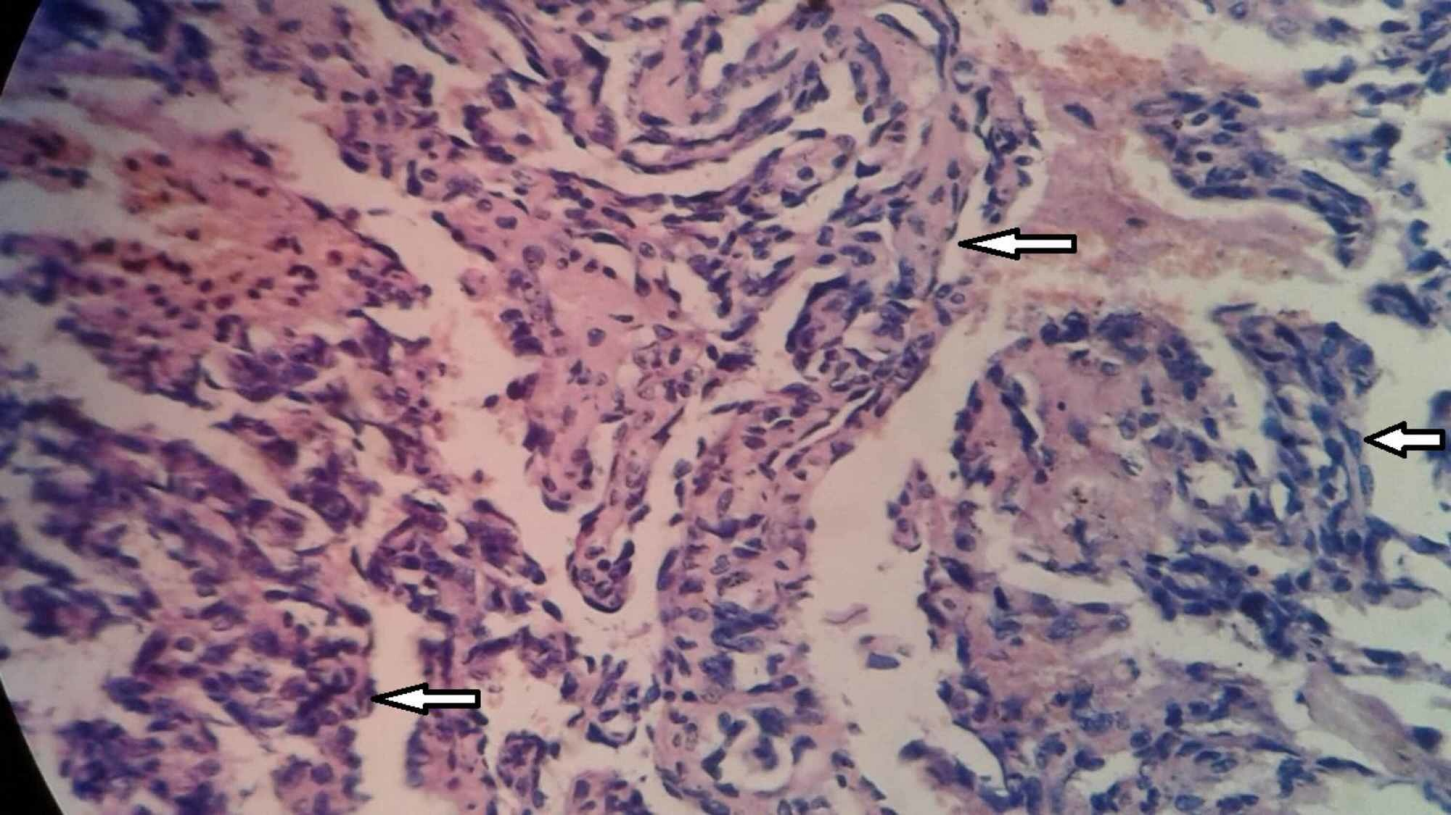
Histopathology showing the presence of freely anastomosing vascular channels lined by atypical endothelial cells (400x, H&E). H&E, hematoxylin and eosin stain

Preoperative histological diagnosis of mammary angiosarcomas by fine needle aspiration cytology (FNAC) or needle biopsy is usually difficult. Chen et al. noted a false negative rate of 37% with needle biopsy [4]. Larger core biopsies might aid in the accurate diagnosis by furnishing a larger sample, but such a macrobiopsy is usually challenging to perform, considering the highly vascular nature of these tumors. Pathologically, breast angiosarcomas are stratified into three groups as per the division by Donnell et al. [13]. Group I (low grade) angiosarcomas contain inter-anastomosing vascular channels coursing through the breast lobules and surrounding ducts. These channels are lined by hyperchromatic endothelial cells and exhibit minimal endothelial tufting. Group II (intermediate grade) tumors in addition to the features of low-grade angiosarcomas exhibit papillary formation with mitoses with or without solid or spindle cell foci. Hemorrhage and necrosis are absent. Group III (high grade) tumors differ from group II by demonstrating prominent endothelial tufting, papillary formation, and solid and spindle cell foci with numerous mitosis, with blood lakes and necrosis (Figures 2-4). The less well-differentiated features of group II and III tumors tend to be located within rather than in the periphery, requiring a thorough review of all sections to stratify these tumors appropriately [13]. This also explains the reason behind the majority of needle biopsies yielding negative results.

 

Although histopathological findings are conclusive, various immunochemical stains for endothelial markers such as CD31, CD34, and factor VIII (von Willebrand factor), and other markers for sarcoma help in finalizing the diagnosis. Amidst these, the most sensitive and specific marker of angiogenic proliferation is CD31 [14-16].

Treatment

Due to the rarity of this tumor, published reports on patients with primary mammary angiosarcoma are limited, making it difficult to establish therapeutic recommendation between total mastectomy and breast-conserving treatment. Complete surgical excision is the key, with a preference given to total mastectomy. Breast-conserving treatment is usually employed in small tumors, where it can serve to be the definitive method of surgical treatment if adequate negative margins can be obtained [10]. Since the tumor metastasizes through the hematogenous route as a rule, lymph node involvement is extremely rare. Hence, routine axillary lymph node dissection is unnecessary unless clinical and intra-operative findings such as enlarged axillary lymph nodes necessitate a radical approach [4].

Owing to mixed results found in various series, there is no international consensus on chemotherapy regimens to date that can be employed in mammary angiosarcomas in the adjuvant setting. The Italian Randomized Cooperative Trail by Frustaci et al. revealed significant improvement in both disease-free survival (DFS) and overall survival (OS) using an adjuvant regimen based on epirubicin plus ifosfamide for adult soft tissue sarcomas [17]. But Sher et al. did not find any survival benefit using anthracycline-ifosfamide or gemcitabine-taxane combination chemotherapy regimens in primary breast angiosarcomas. However, given the activity of cytotoxic chemotherapy in metastatic mammary angiosarcomas, adjuvant chemotherapy should be unquestionably advocated in patients with high risk localized breast angiosarcomas [18].

Adjuvant radiotherapy has been used in breast sarcomas with the aim of enhancing both locoregional control after surgery as well as the OS. This treatment modality is particularly valuable in patients with microscopically positive (R1) margins. In the retrospective study conducted by Johnstone et al. to determine the efficacy of adjuvant radiotherapy in primary mammary sarcomas, it was found that radiotherapy provided excellent local control with an actuarial five year DFS and OS of 68% and 66%, respectively [19]. Even though the result seems to be encouraging, this lacks validity due to the small sample size.

Prognosis

Prognosis of primary mammary angiosarcoma such as other soft tissue sarcomas is dependent on tumor size, grade, and resection margin status [16]. While the interface between tumor grade and margin status with prognosis has been clearly established, the literature presents contrasting opinions regarding the relationship between tumor size and prognosis. Authors like Rosen et al., Bousquet et al., and Blanchard et al. in their observations found no association between the primary tumor size and the DFS or OS [5,20,21]. However, authors like Sher et al. and Adem et al. found contradictory results in their series of primary mammary sarcomas, proving the association of tumor size with DFS and OS [18,22]. Zelek et al. in their study on prognostic factors for primary breast angiosarcomas also found a strong association between the tumor size and DFS, without any significant influence on OS [23].

## Conclusions

Primary angiosarcoma of breast is an extremely rare and aggressive malignancy affecting young females, usually presenting with a rapidly growing painless mass. When breast imaging shows a large vascular mass, odds of angiosarcoma should be considered. Diagnosis should be confirmed with core needle biopsy, though it is associated with a significant false-negative rate. Complete surgical excision is the key, with adjuvant chemotherapy in high-risk patients and radiotherapy particularly valuable in cases with microscopically positive margins, to improve the locoregional control and survival. Despite the aggressive multimodal therapy, prognosis remains poor.
